# Supplementary material for: Genome-wide association meta-analysis for early age-related macular degeneration highlights novel loci and insights for advanced disease
Source: BMC Med Genomics. 2020 Aug 26;13:120. doi: 10.1186/s12920-020-00760-7 (PMC7449002; doi:10.1186/s12920-020-00760-7)
Supplement: Supplementary file 2 — Additional file 2: Supplementary Note. [file 12920_2020_760_MOESM2_ESM.docx]

**Supplementary Note 1. Performance of the automated classification in UK Biobank**

We evaluated the performance of the automated disease classification by selecting 2,013 individuals (4,026 fundus images) for manual classification based on the 3CC Severity Scale [1]. We found a concordance of 79.5% (**Table S3**). When measuring the concordance of the automated and the manual classification with a Cohen’s Kappa statistic, we found a kappa of 0.61; when computing the Kappa statistics after list-wise exclusion of ungradable individuals (i.e. individuals with missing AMD status either in the manual or in the automated classification) [2], we found a kappa of 0.47. The regular kappa is considered biased towards the null in the case of missings; the kappa using list-wise exclusion of missings is considered less biased, in the case of missingness-at-random [2]. We do not find a larger kappa after list-wise exclusion compared to the regular Kappa.

One may consider that self-reported AMD or ICD10-code based AMD could be used to identify individuals with AMD (“Macular degeneration” in UKBB data field 6148 and “H35.3: Degeneration of macula and posterior pole” data field 41270, respectively). Since both provide only a binary outcome (AMD yes/no), this is not suitable to be used for an early AMD GWAS. Still, one may ask the question how the automated AMD classification compares to self-reported or ICD10-code based AMD, when focusing on any AMD. We thus compared the automatically derived AMD status for the 55,475 individuals in the full UK Biobank with self-reported AMD and AMD from ICD-10 codes (**Table S4**). After list-wise exclusion of individuals with missing information and collapsing early and late AMD to any AMD, we found a kappa of 0.12 and 0.025 comparing the automated classification with self-report and ICD-10 code, respectively.

**References**

1. Guenther F, Brandl C, Winkler TW, Wanner V, Stark K, Kuechenhoff H, et al. Chances and challenges of machine learning based disease classification in genetic association studies illustrated on age-related macular degeneration. bioRxiv. 2019;

2. De Raadt A, Warrens MJ, Bosker RJ, Kiers HAL. Kappa Coefficients for Missing Data. Educ. Psychol. Meas. 2019;
