# Supplementary material for: Genome-wide association meta-analysis for early age-related macular degeneration highlights novel loci and insights for advanced disease
Source: BMC Med Genomics. 2020 Aug 26;13:120. doi: 10.1186/s12920-020-00760-7 (PMC7449002; doi:10.1186/s12920-020-00760-7)
Supplement: Supplementary file 3 — Additional file 3: Supplementary Figures. [file 12920_2020_760_MOESM3_ESM.docx]

**SUPPORTING FIGURES**

**Figure S1. CD46 regional association.** Shown are regional association plots for the *CD46* locus (A: primary meta-analysis, B: Approximate conditional GCTA analyses, conditioned on rs4844620). Color indicates the correlation (r²) to rs4844620.

**
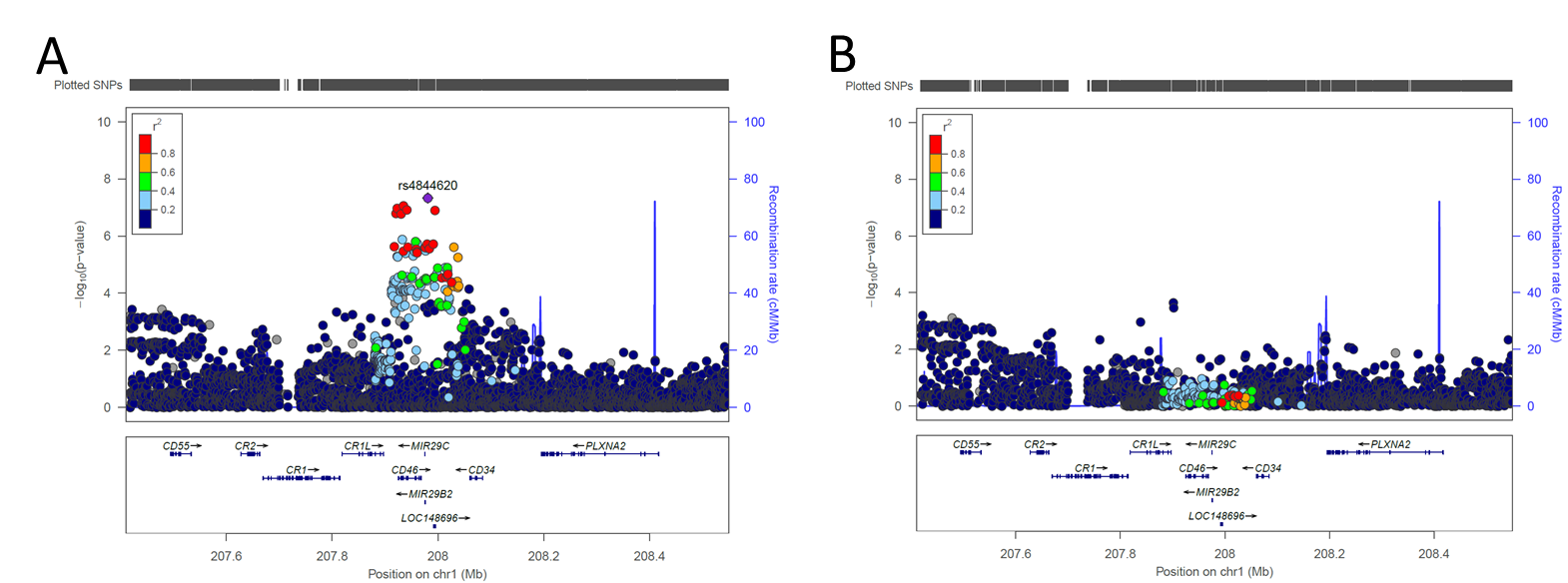
**

**Figure S2. TYR regional association.** Shown are regional association plots for the *TYR* locus (A: primary meta-analysis, B: Approximate conditional GCTA analyses, conditioned on rs621313). Color indicates the correlation (r²) to rs621313.


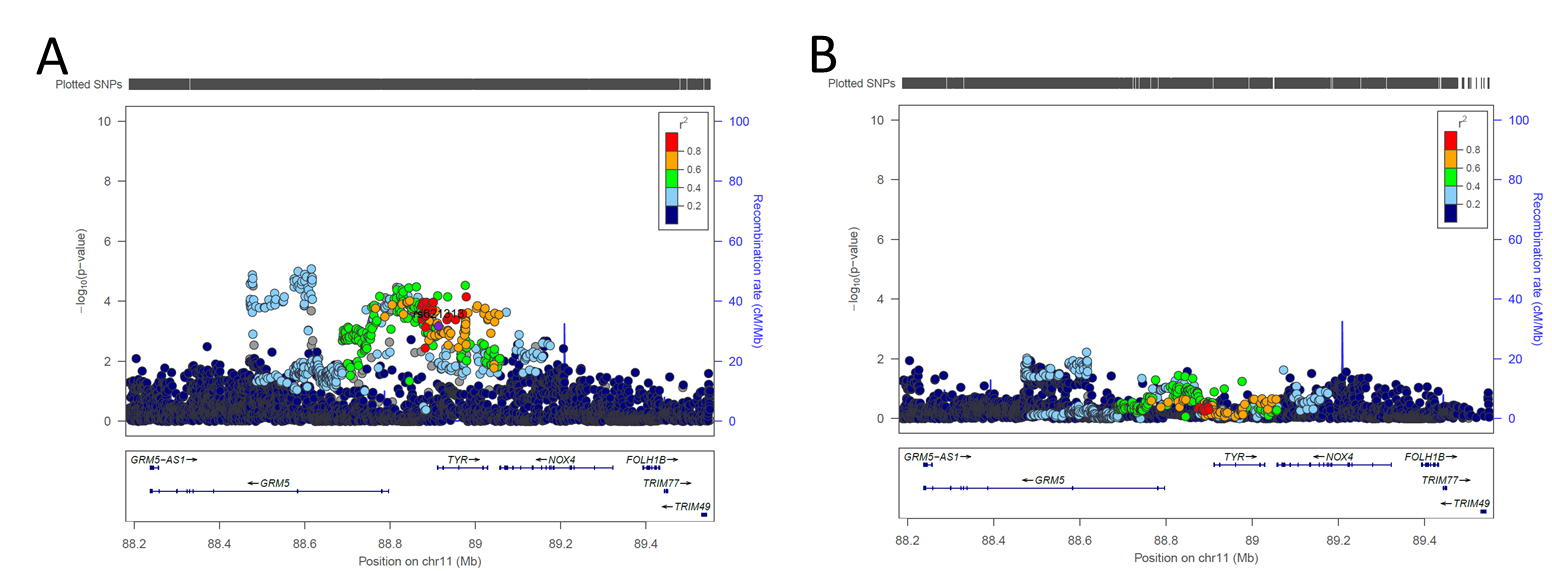


**Figure S3. Leave-one-out meta-analysis and study-specific results for the 10 identified loci.** The forest plots show log odds ratios on early AMD with confidence intervals from each of the 11 leave-one-study-out meta-analyses (left forest plot; the first column states the excluded study) and from the study-specific GWAS (right forest plot). We show results for the eight lead variants identified from the genome-wide approach (confidence intervals shown for α=5x10^-8^; A: rs4844620 near *CD46*, B: rs547154 near *C2*, C: rs943080 near *VEGFA*, D: rs13278062 near *TNFRSF10A*, E: rs5817082 near *CETP*, F: rs11569415 near *C3*, G: rs4658046 near *CFH*, H: rs3750847 near *ARMS2/HTRA1*) and for the two additional lead variants identified by the candidate approach (confidence intervals shown for α=0.05/14; I: rs621313 near *TYR*, J: rs6857 near *APOE*).

**
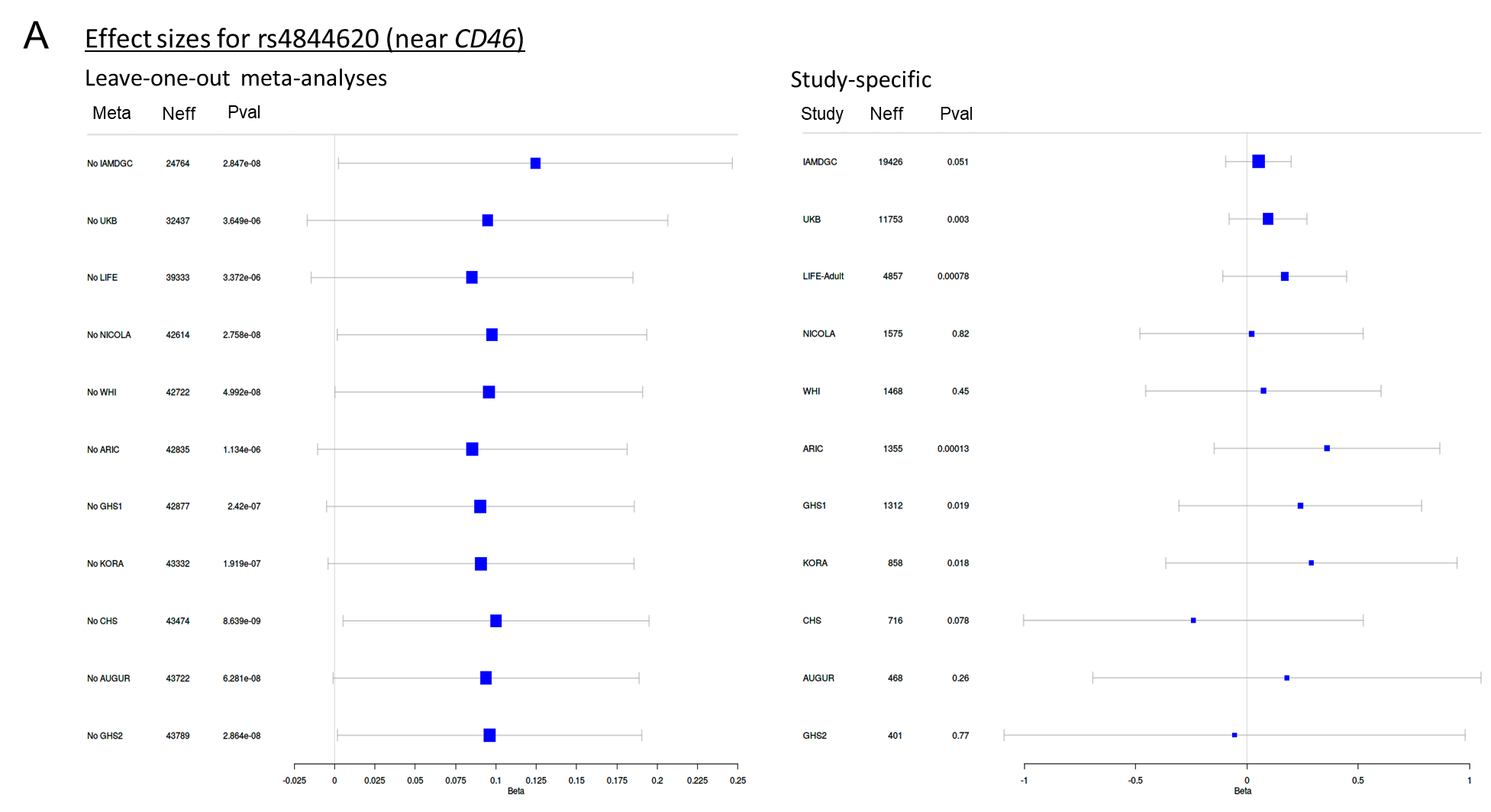
**

**
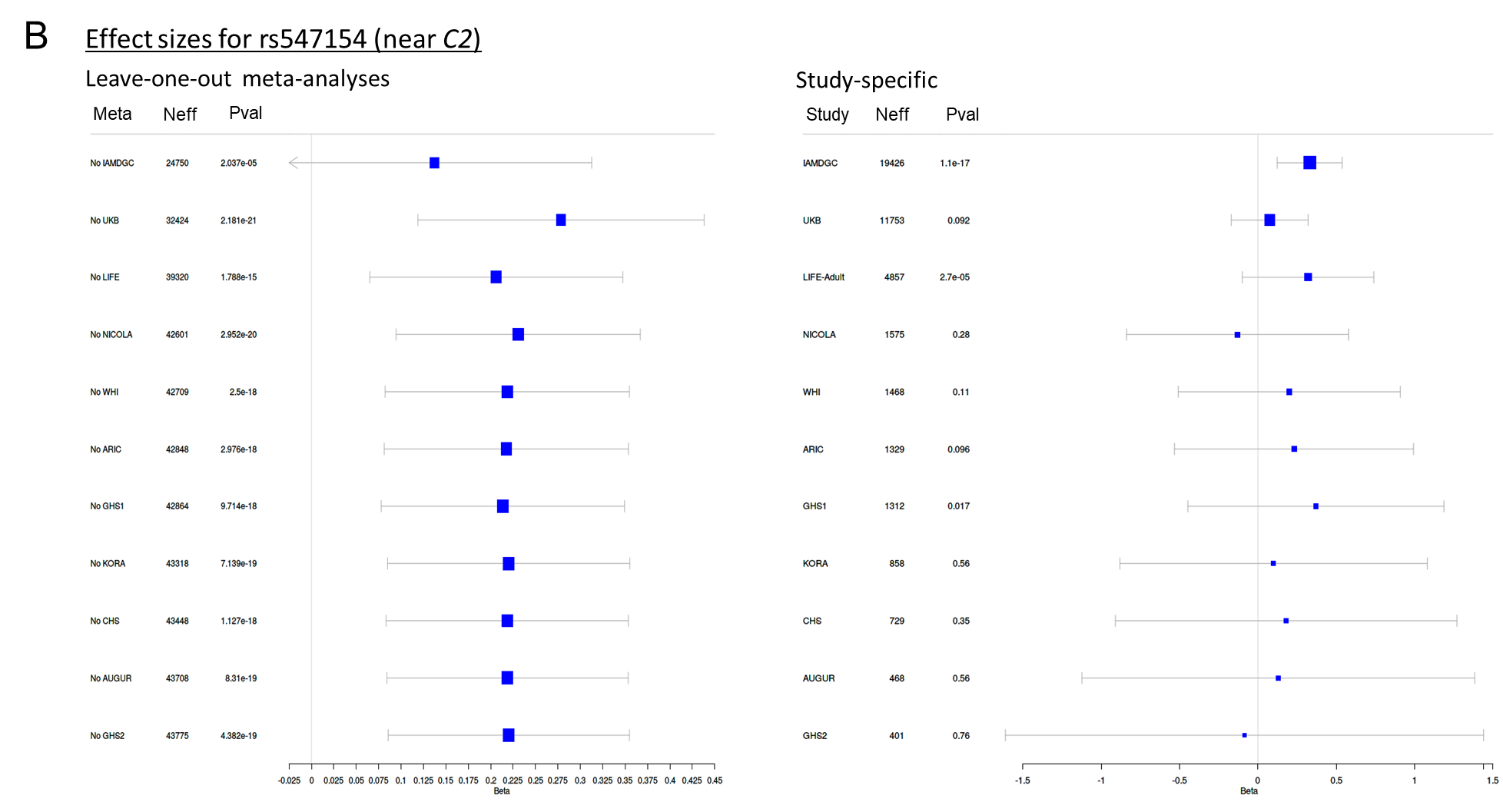
**

**
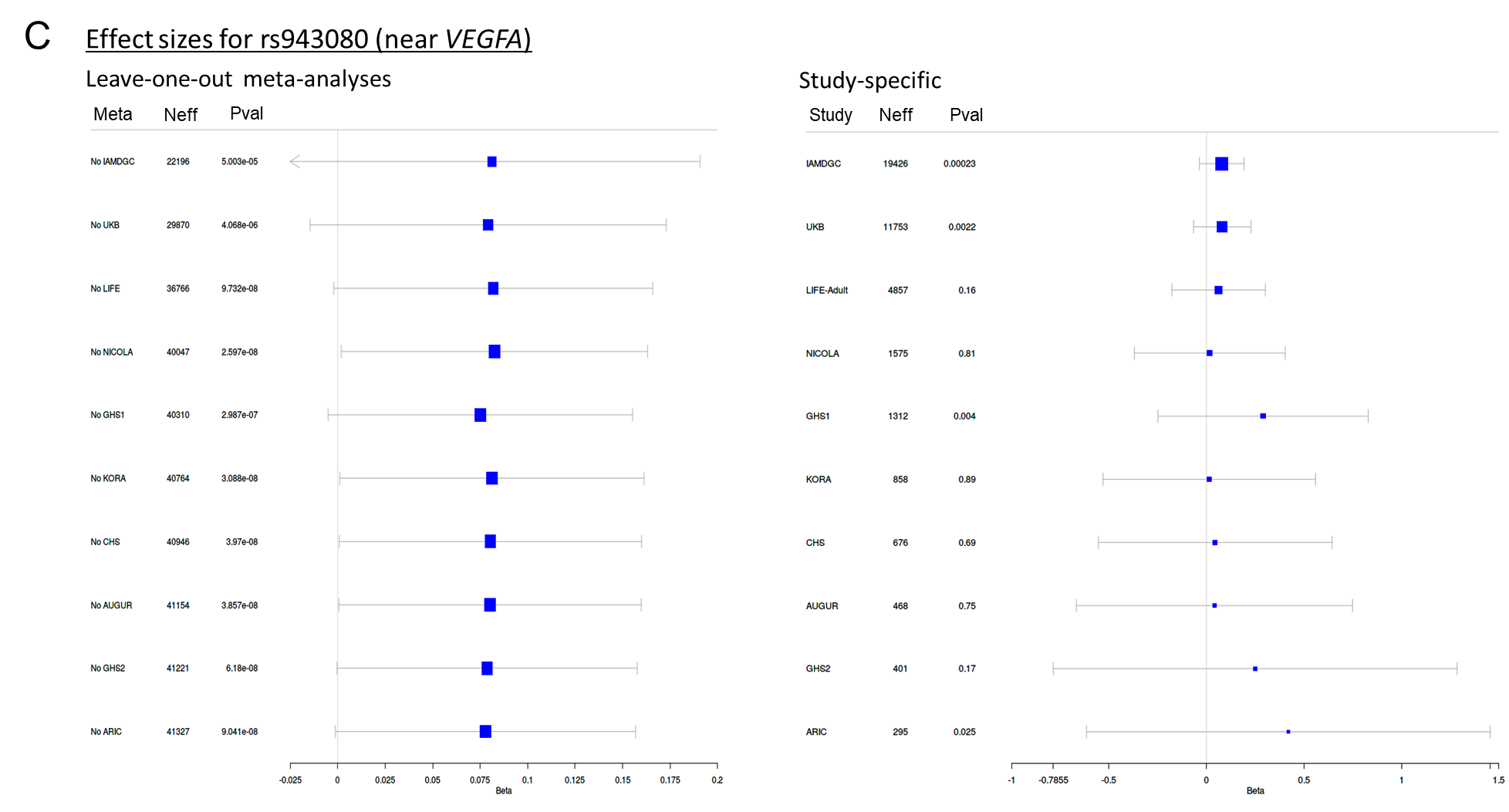
**

**
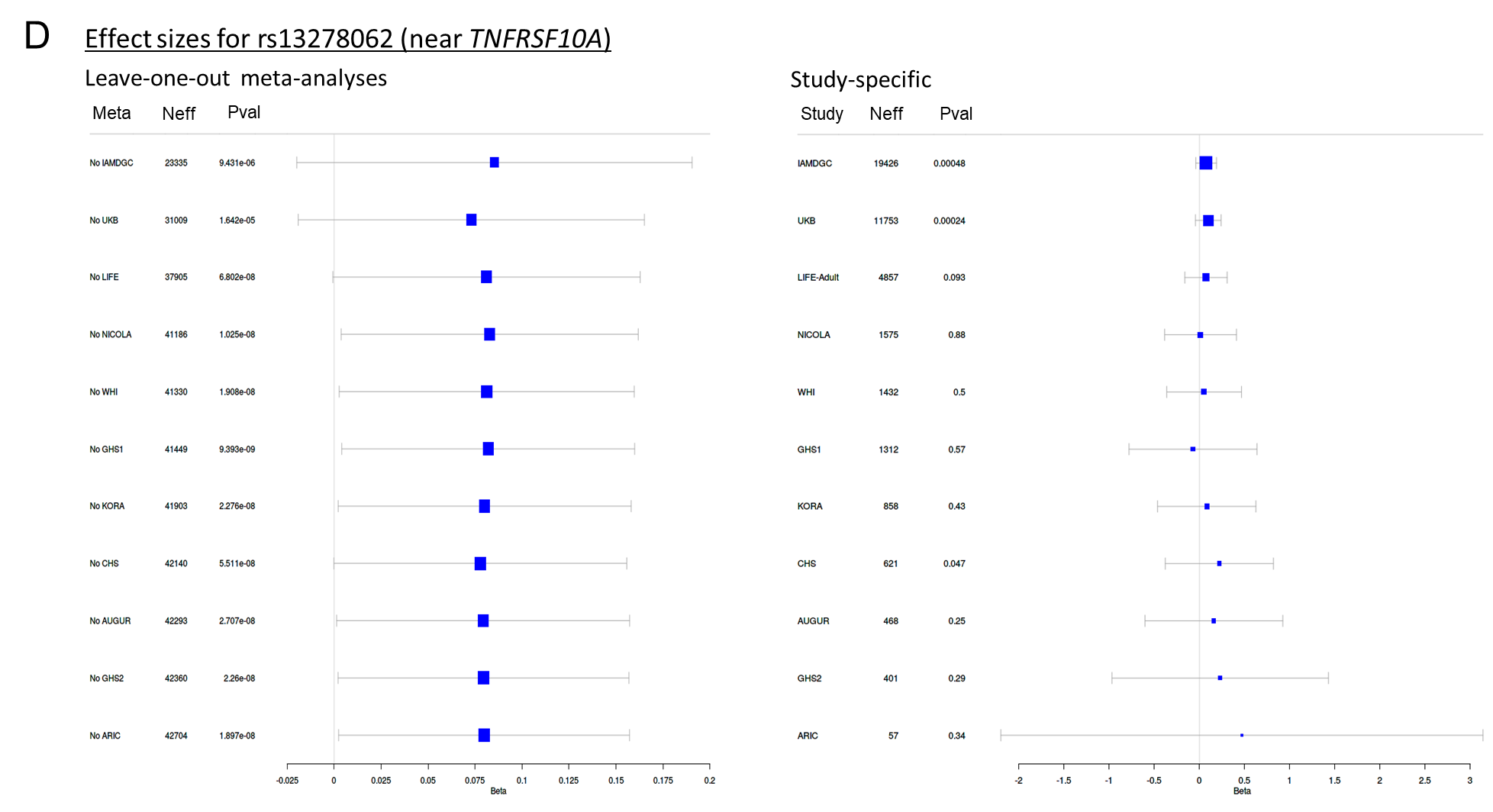
**

**
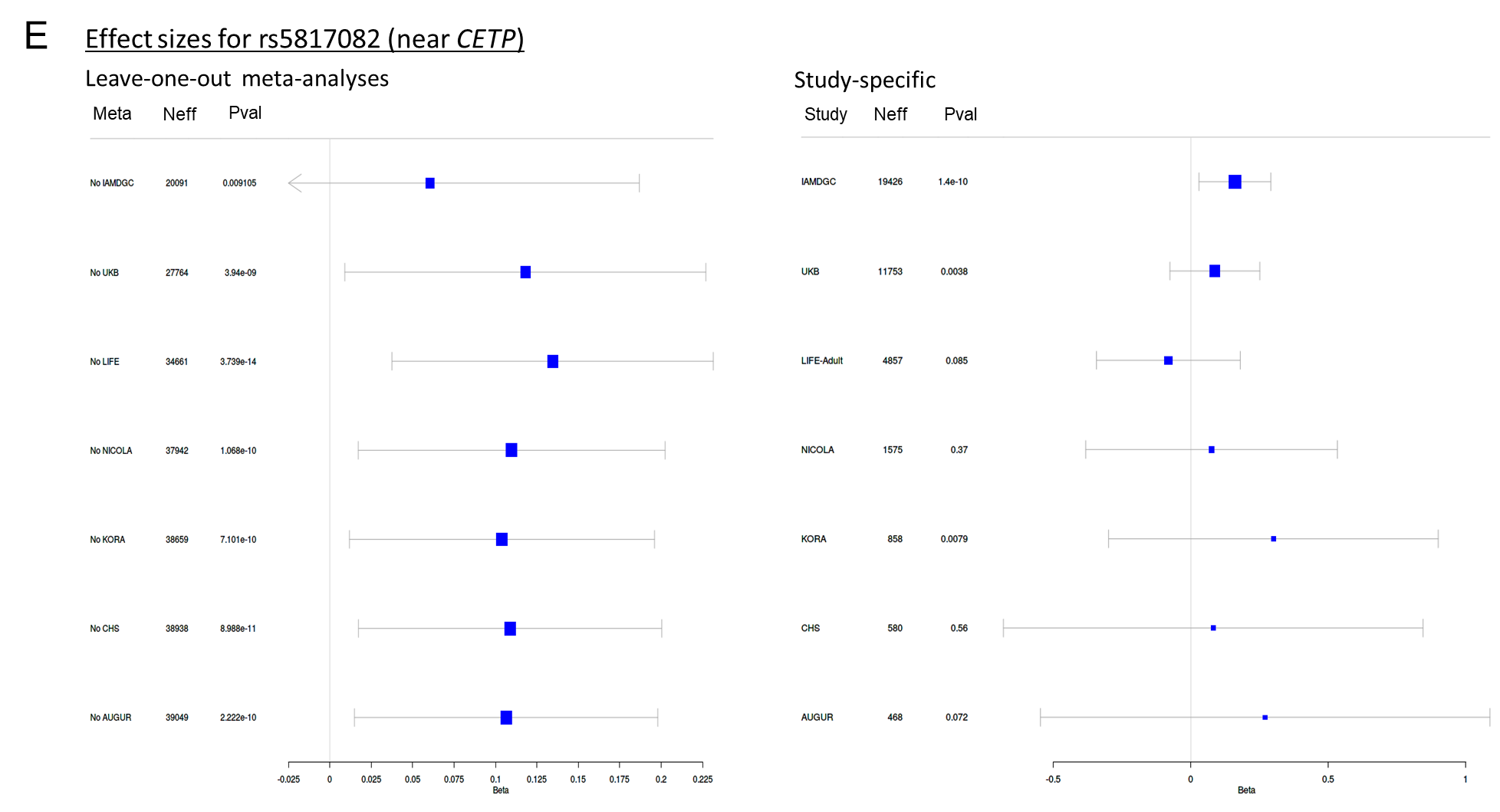
**

**
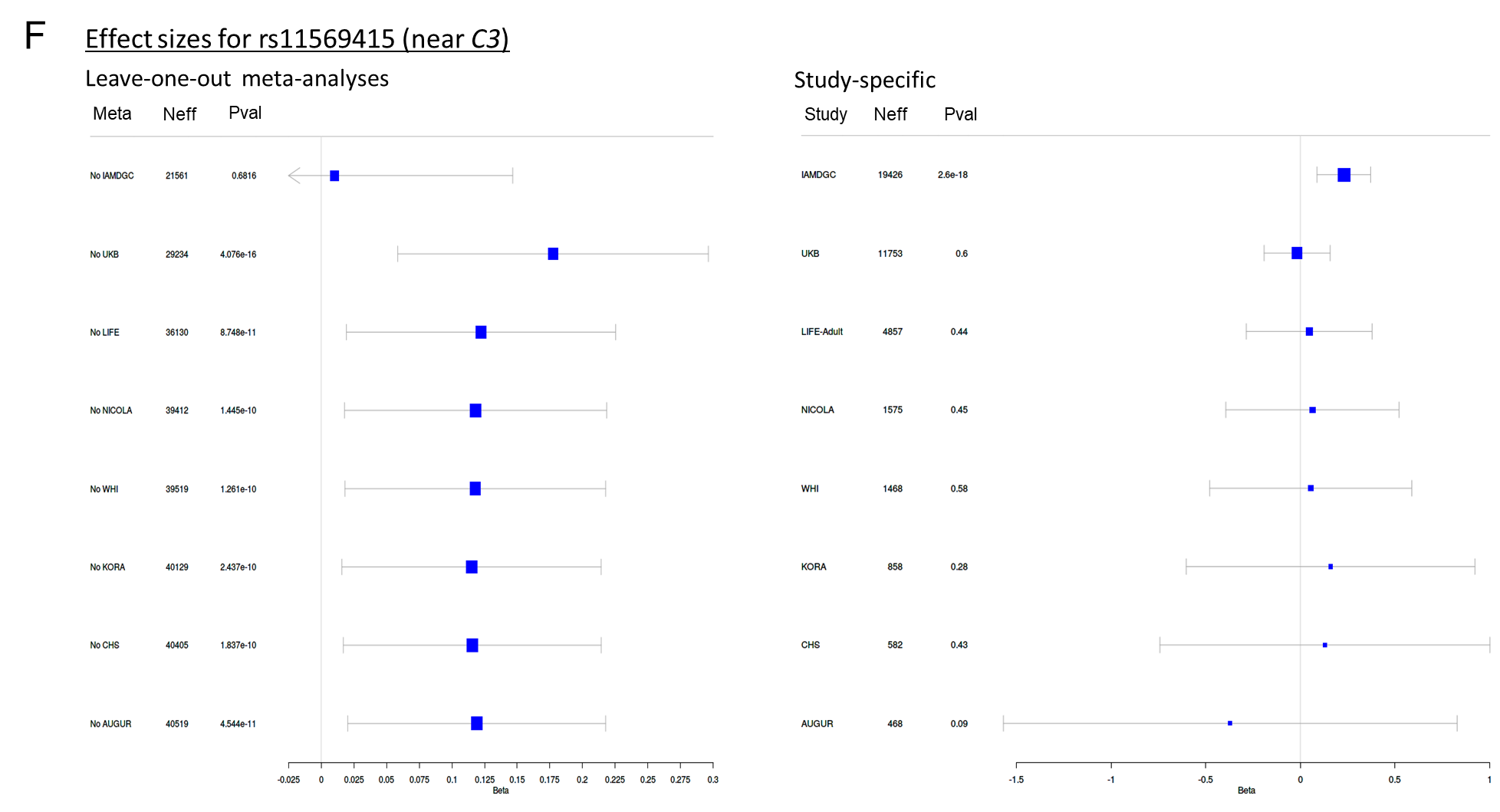
**

**
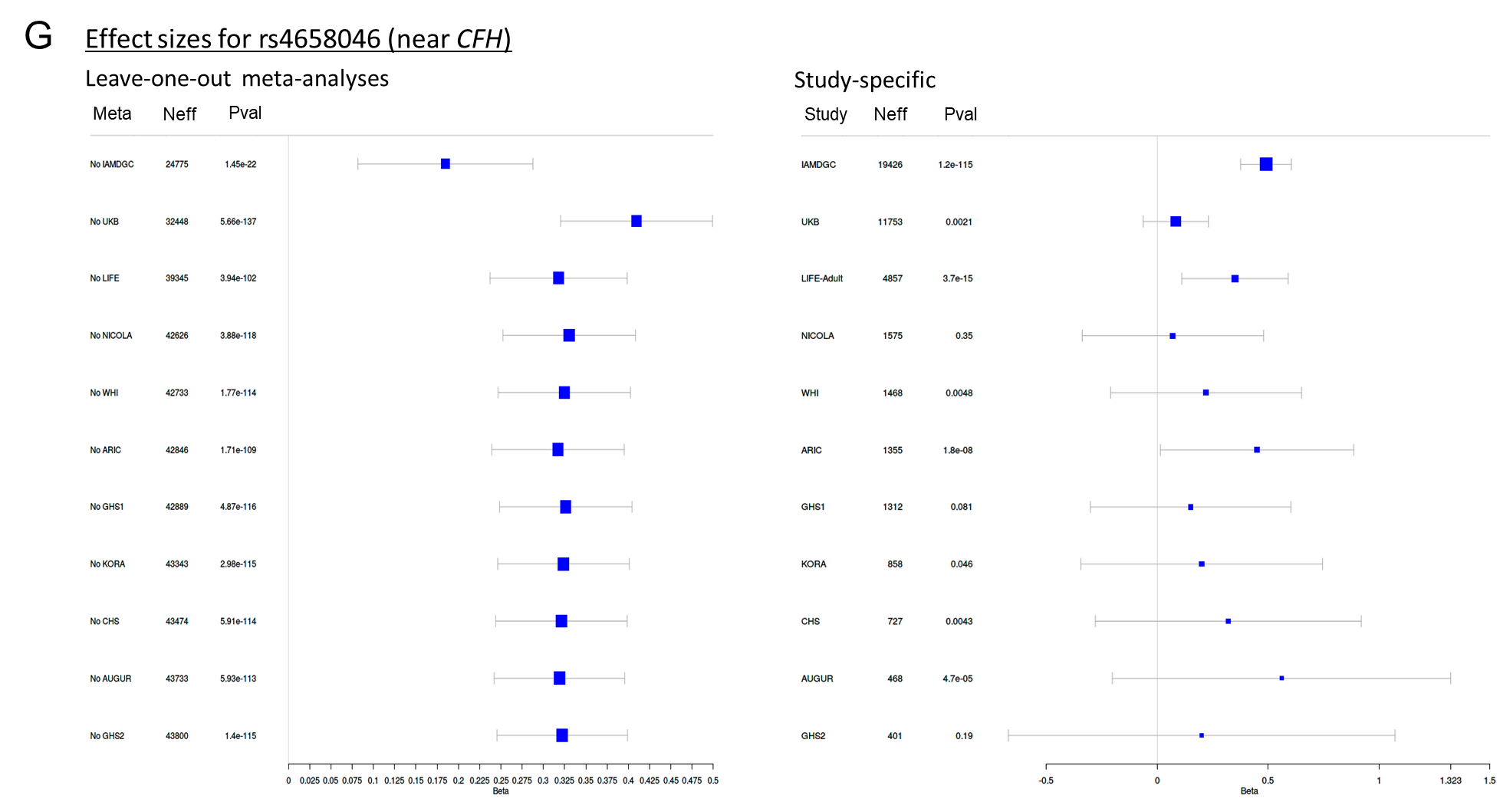
**

**
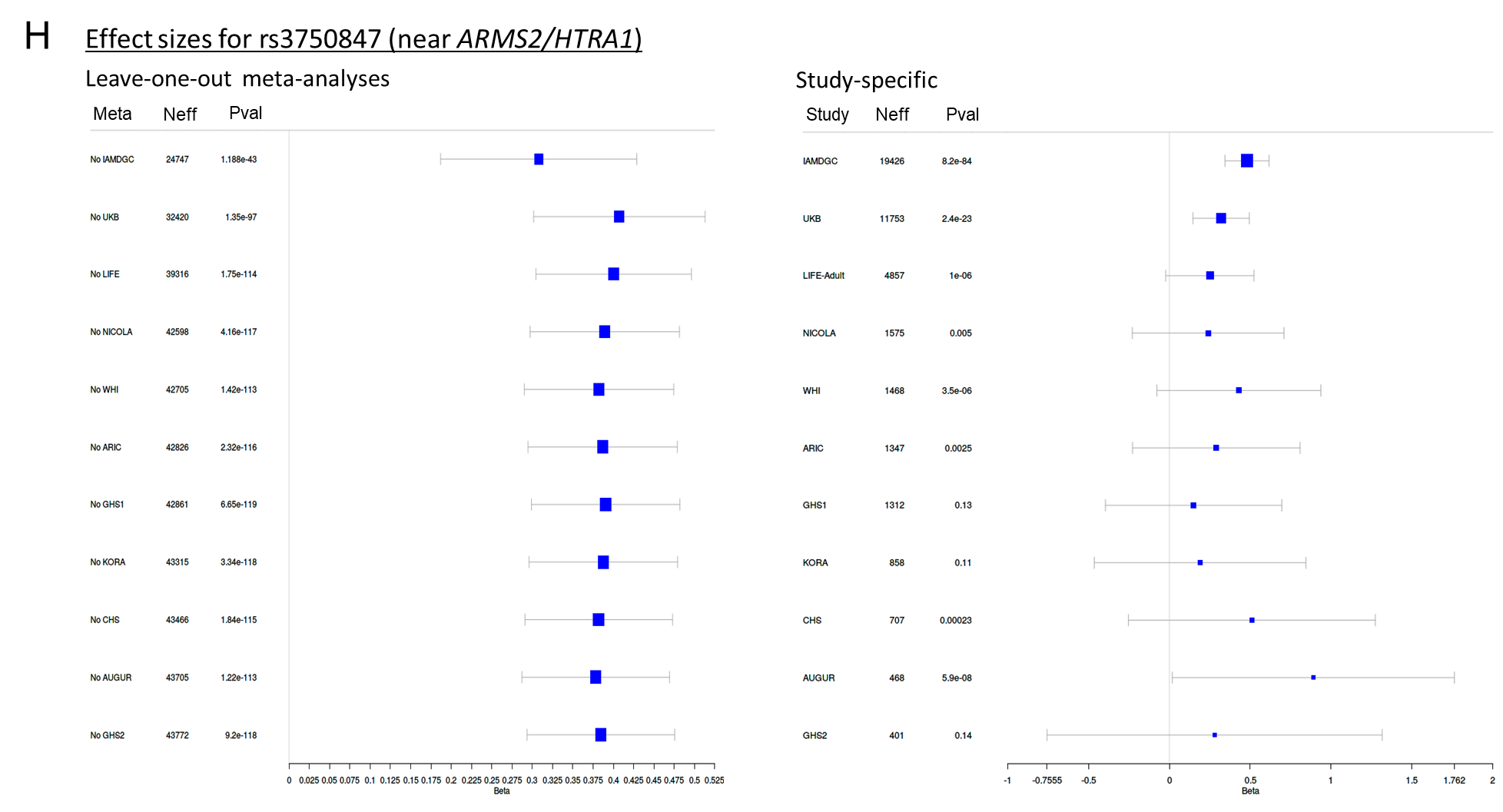
**

**
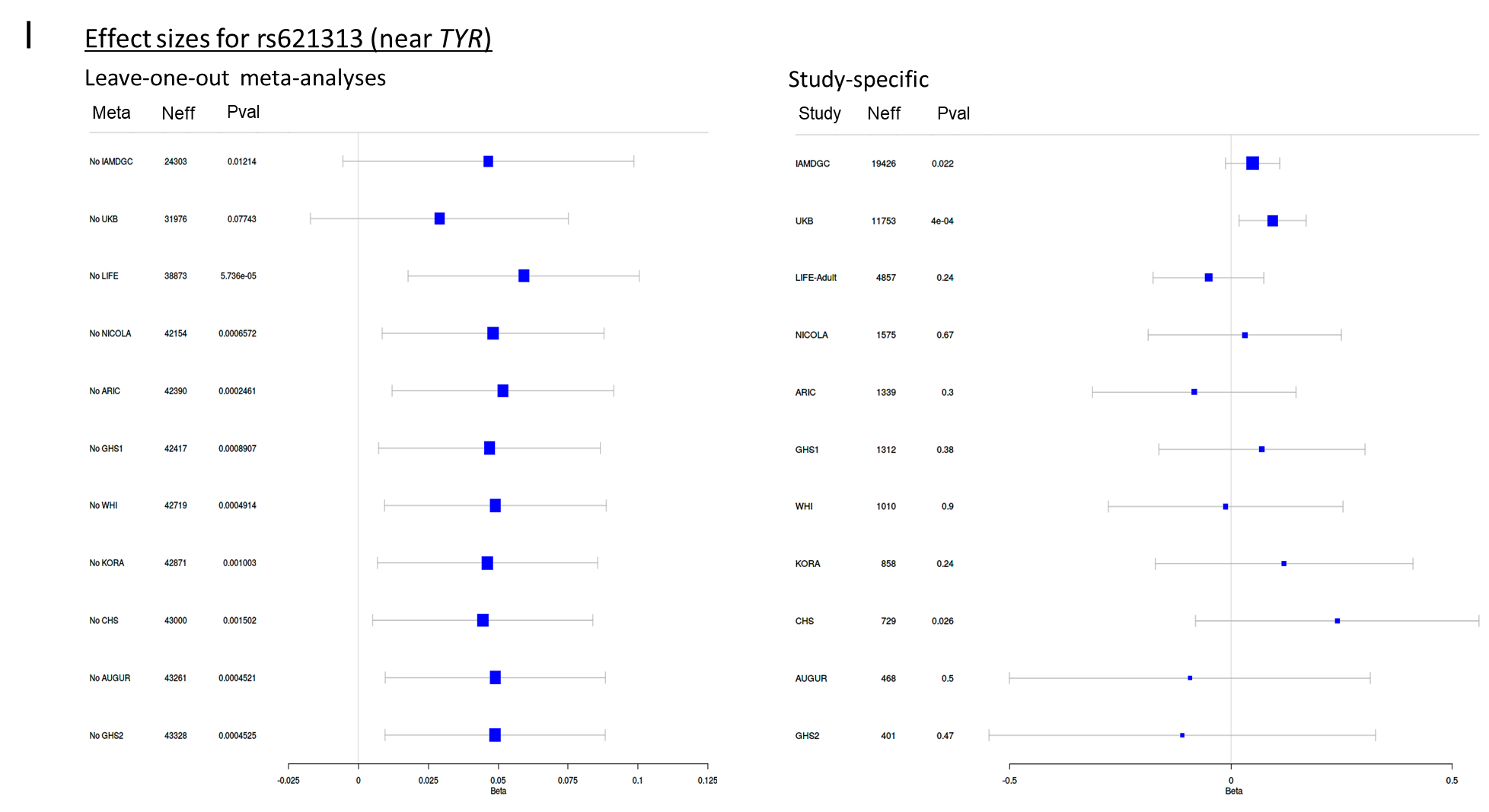
**

**
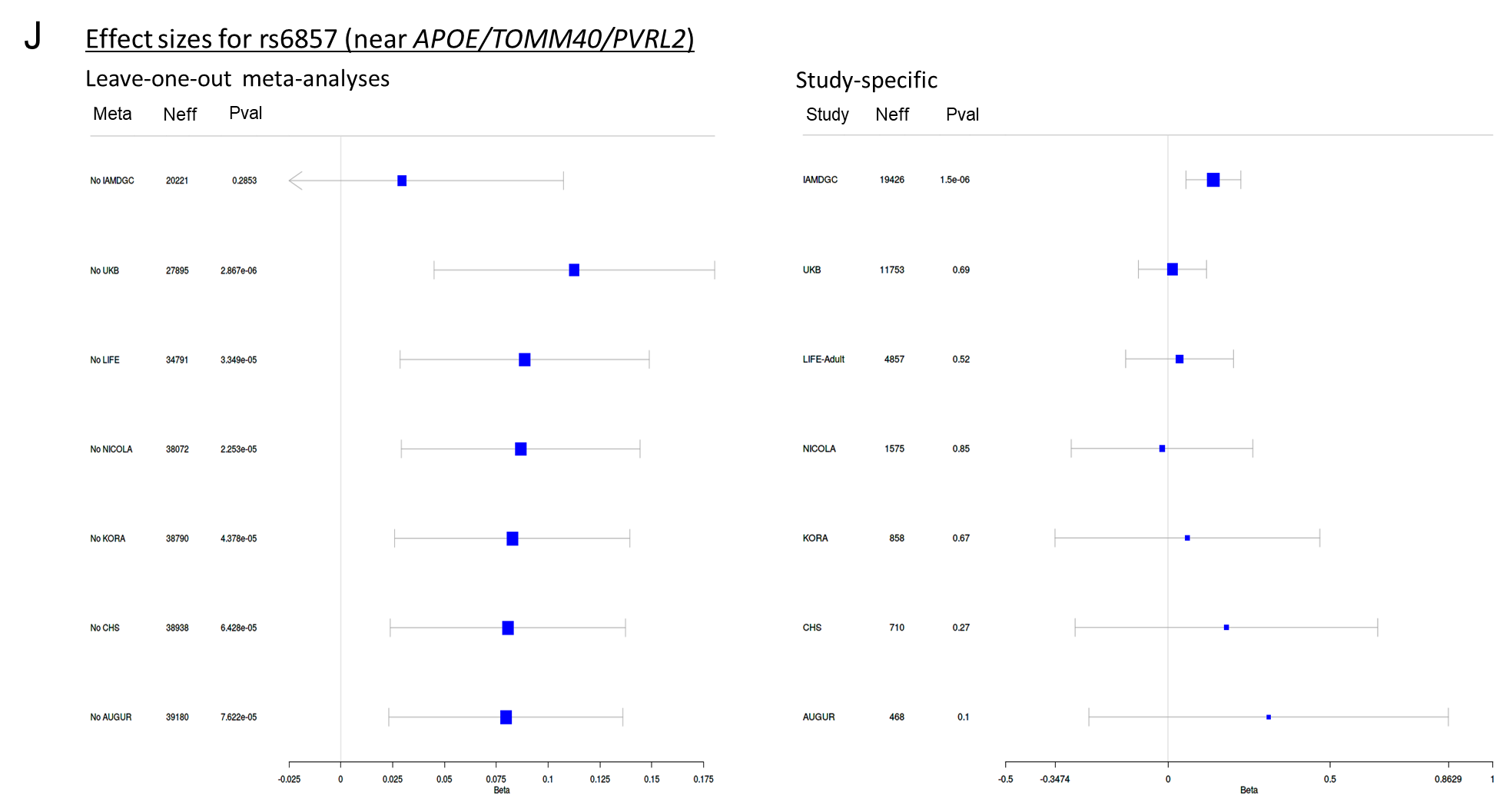
**

**Figure S4. Sensitivity analysis for including additional covariates.** The figure shows results from sensitivity regression analysis for the 10 identified variants (red: *CD46* lead variant from GWAS; blue: *TYR* variant from candidate approach) in IAMDGC (A-C) and UKBB study data (E-F). We compared our original early AMD effect sizes that were adjusted for age and 2 principal components (PCs, and also for whole genome amplification status in IAMDGC) with effect sizes from three sensitivity analyses: (A,D): additional adjustment for sex, (B,E): additional adjustment for eight further PCs, (C,F): additional adjustment for sex and eight further PCs.

**
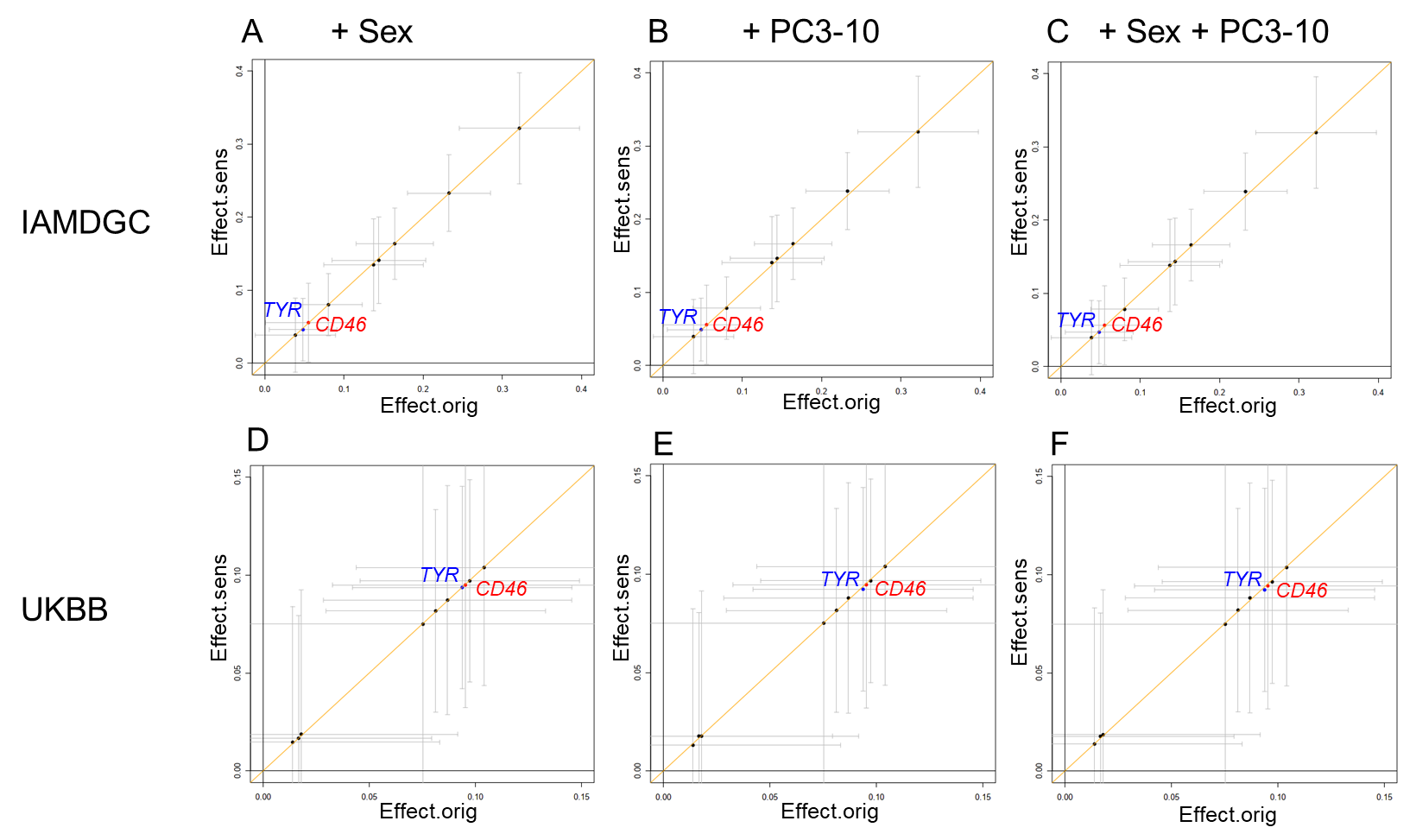
**

**Figure S5.** Colocalization of early AMD association and expression of *CD46* or *CD55* in human retina. The scatter plots compare association P values for early AMD (x axis) with association on expression of CD46 (panel A, y axis) or CD55 (panel B, y axis). The variants are colored by r^2^ to the *CD46* lead variant rs4844620 (purple triangle).

**
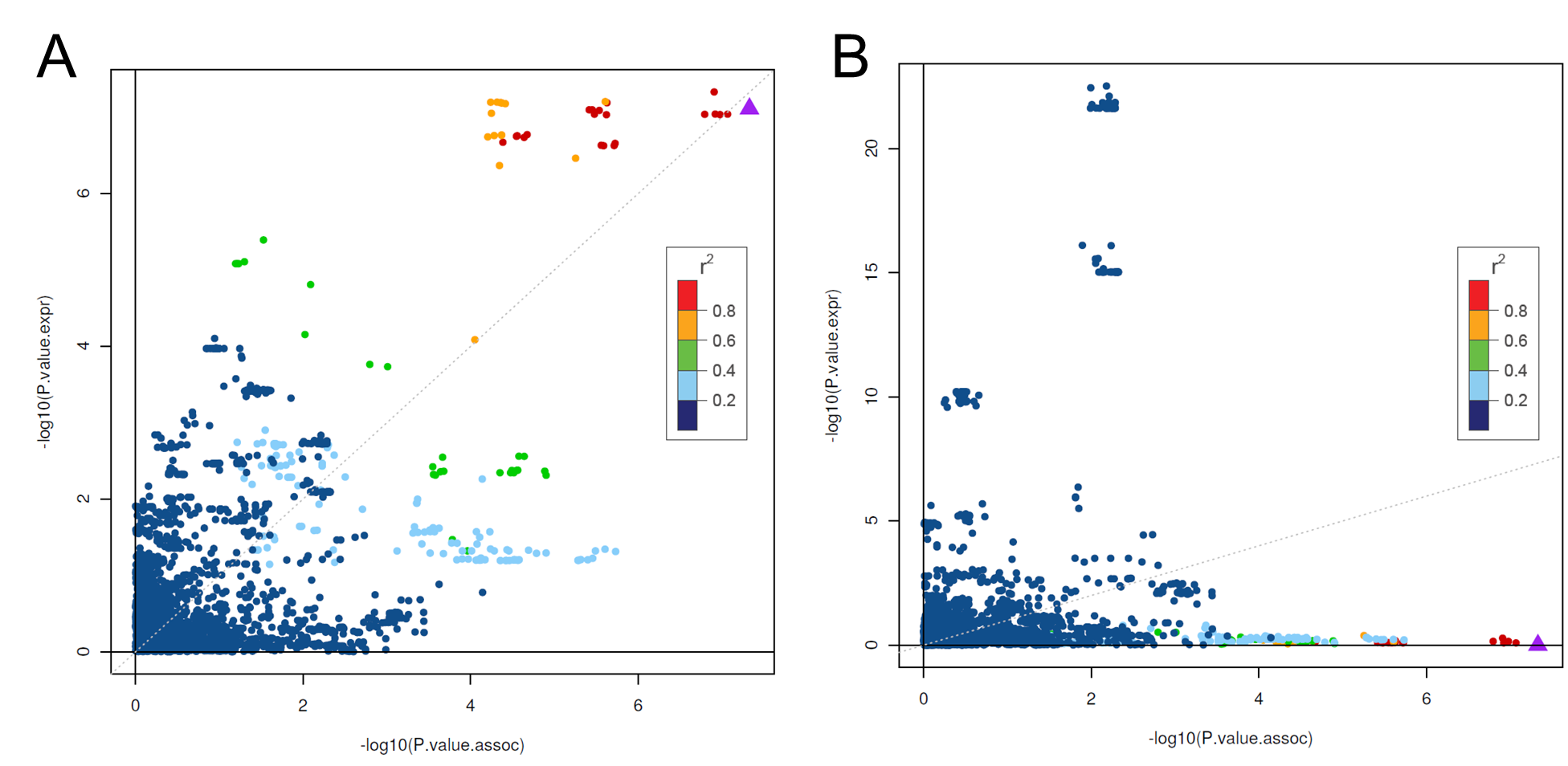
**

**Figure S6.** Colocalization of association on early AMD (meta-analysis) and expression of *CD46, CD34, PLXNA2* and *CD55* in human retina. The plots compare the association P values for expression (darkred: no significant effect on expression, red: significant effect on expression) with early AMD (blue) by chromosomal base position (x axis).


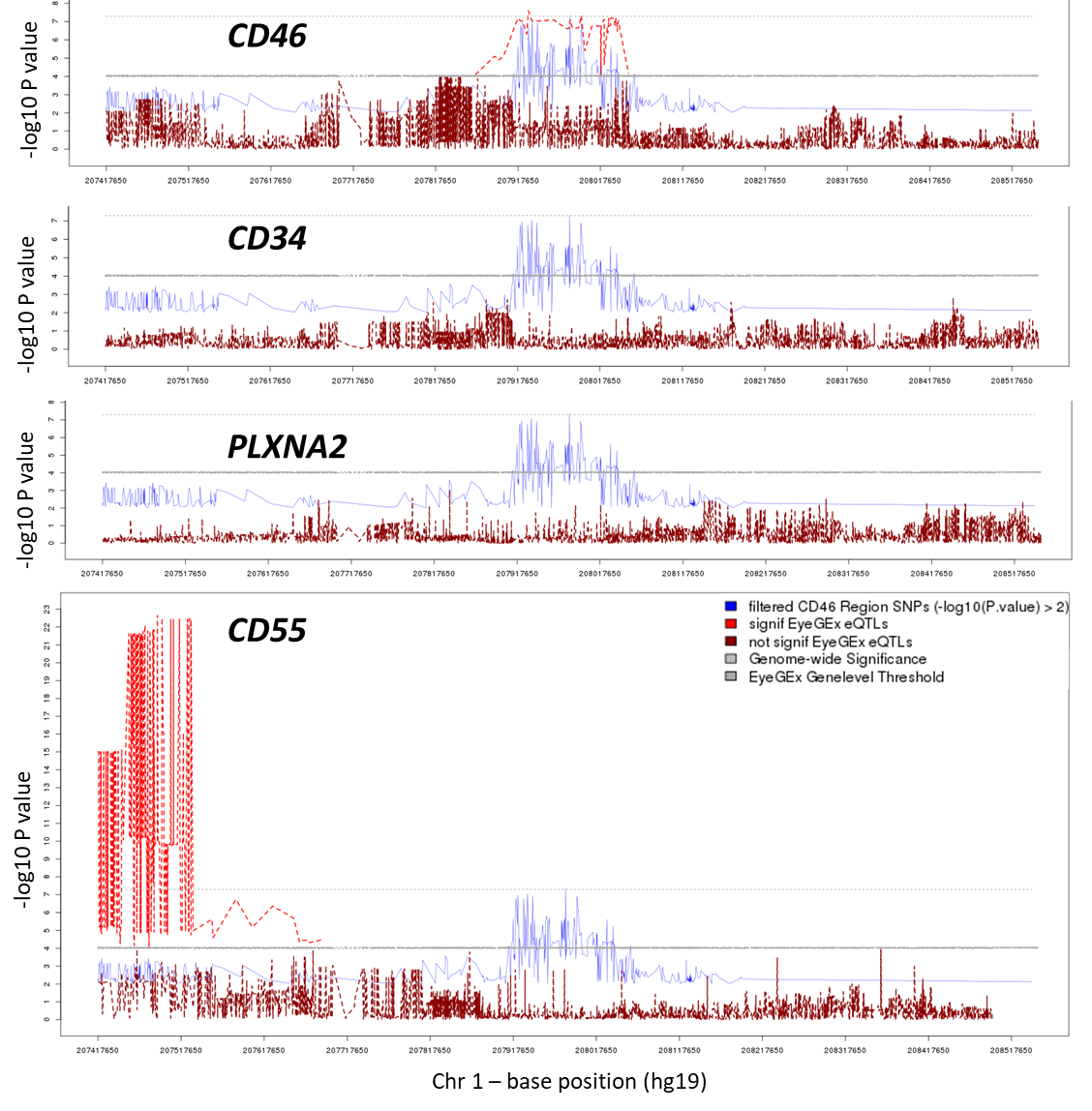


**Figure S7.** Expression of candidate genes at the *CD46* locus in EyeIntegration data for Fetal Retina (A), Adult Retina (B), Fetal RPE (C) and Adult RPE (D). The percentiles denote the distribution of gene expression across all genes (approximately 36,852 genes measured) in the respective tissue.


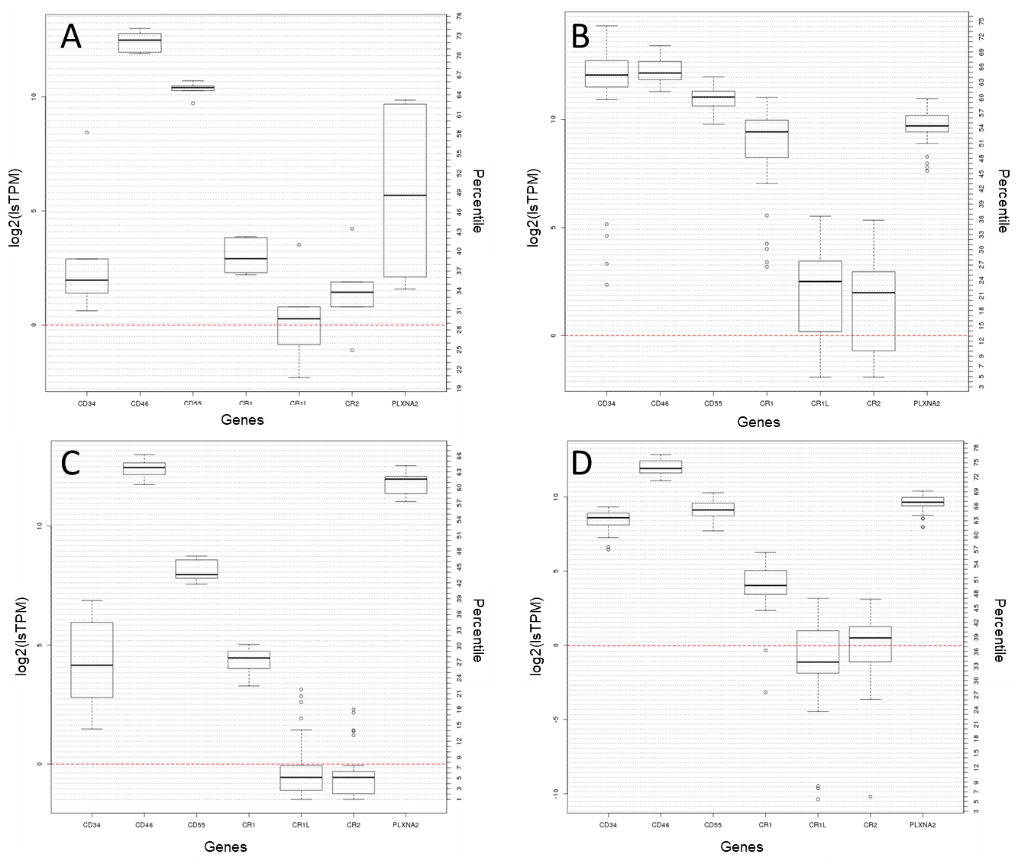


**Figure S8.** Expression of candidate genes at the *TYR* locus in EyeIntegration data for Fetal Retina (A), Adult Retina (B), Fetal RPE (C) and Adult RPE (D). The percentiles denote the distribution of gene expression across all genes (approximately 36,852 genes measured) in the respective tissue.

**
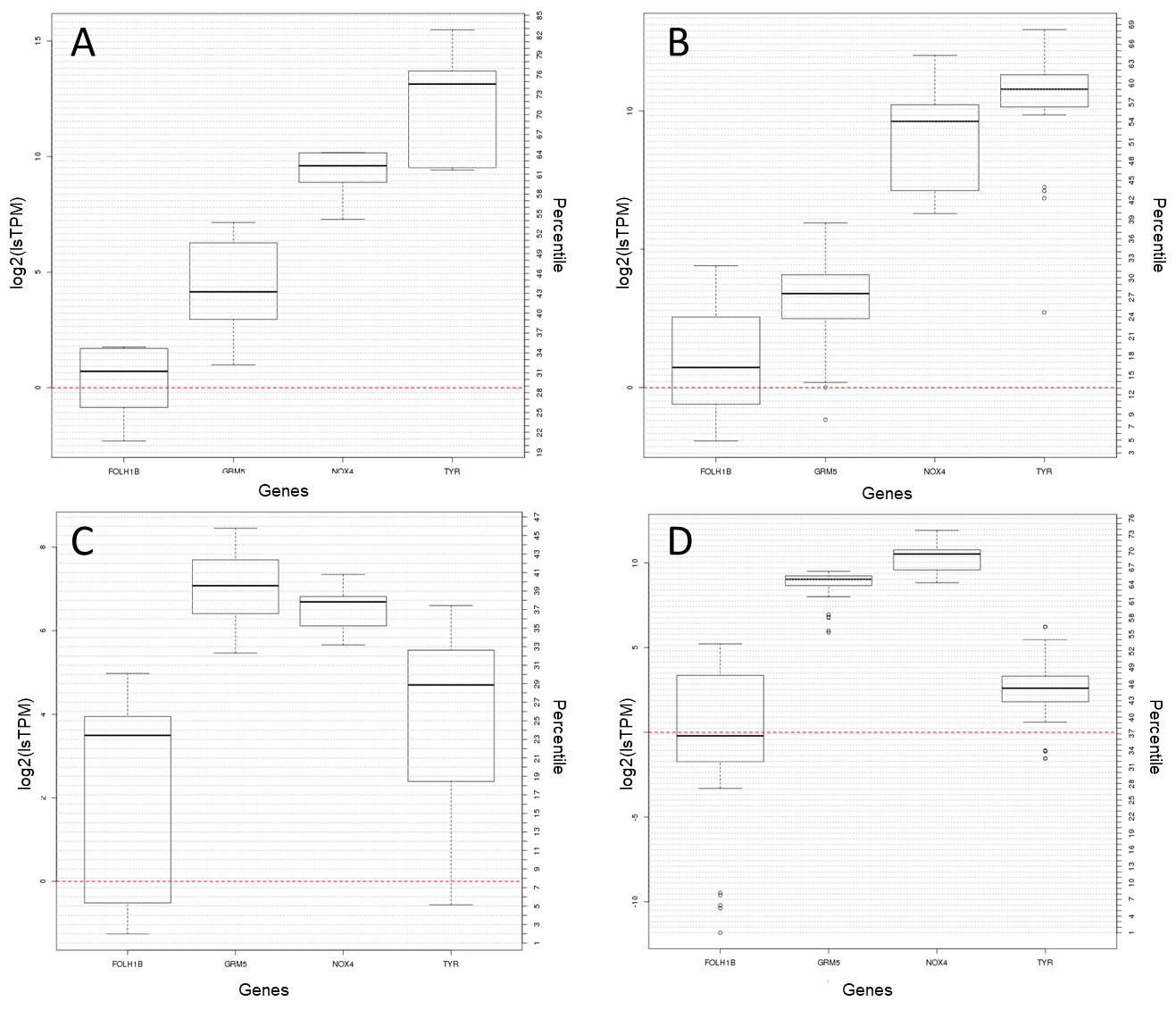
**
